# Chronic lymphedema in patients with kaposiform hemangioendothelioma: incidence, clinical features, risk factors and management

**DOI:** 10.1186/s13023-020-01595-2

**Published:** 2020-11-07

**Authors:** Yi Ji, Siyuan Chen, Chuncao Xia, Jiangyuan Zhou, Xian Jiang, Xuewen Xu, Kaiying Yang, Xuepeng Zhang, Feiteng Kong, Guoyan Lu, Yongbo Zhang

**Affiliations:** 1 Division of Oncology, Department of Pediatric Surgery, West China Hospital of Sichuan University, Chengdu, 610041 China; 2 Pediatric Intensive Care Unit, Department of Critical Care Medicine, West China Hospital of Sichuan University, #37# Guo-Xue-Xiang, Chengdu, 610041 China; 3 Department of Radiology, West China Hospital of Sichuan University, Chengdu, 610041 China; 4 Department of Dermatology, West China Hospital of Sichuan University, Chengdu, 610041 China; 5 Department of Burn and Plastic Surgery, West China Hospital of Sichuan University, Chengdu, 610041 China; 6 Department of Pediatric Surgery, Sichuan Women and Children’s Hospital, Chengdu, 610045 China; 7 Pediatric Intensive Care Unit, West China Second University Hospital, Sichuan University, Chengdu, 610041 China; 8 Department of Pediatric Surgery, Chengdu Women and Children’s Central Hospital, Chengdu, 610031 China

**Keywords:** Kaposiform hemangioendothelioma, Kasabach–Merritt phenomenon, Clinical characteristics, Complication, Risk factor

## Abstract

**Objectives:**

There are no cohort studies of chronic lymphedema in patients with kaposiform hemangioendothelioma (KHE). We sought to characterize the incidence, clinical features, risk factors and management of chronic lymphedema in patients with KHE.

**Methods:**

We conducted a multicenter retrospective analysis of patients who had a minimum of 3 years of follow-up after the onset of KHE and/or Kasabach–Merritt phenomenon (KMP). Clinical features were reviewed to determine the possible cause of chronic lymphedema. The degree of lymphedema, risk factors and management strategies were analyzed.

**Results:**

Among the 118 patients, chronic lymphedema was confirmed by lymphoscintigraphy 1 year after the onset of KHE and/or KMP in 13 patients. In 8 patients with lymphedema, extremity swelling was evident in the presence of KHE and/or KMP. In all patients with lymphedema, a unilateral extremity was affected, along with ipsilateral KHE. Most (84.6%) patients reported moderate lymphedema. Lymphedema was more common in patients with larger (≥ 10 cm) and mixed lesions involving the extremities (*P* < 0.01). A history of KMP and sirolimus treatment were not predictors of lymphedema (*P* > 0.05). Overall, 76.9% of patients received sirolimus treatment after referral, including 53.8% who presented extremity swelling before referral. Seven (53.8%) patients received compression therapy. Five (38.5%) patients reported lymphedema-associated decreased range of motion at the last follow-up.

**Conclusions:**

Chronic lymphedema is a common sequela of KHE and can occur independently of KMP and sirolimus treatment. Patients with large and mixed KHE involving extremities should be closely monitored for this disabling complication.

## Introduction

Lymphedema is a localized form of tissue swelling due to anomalies and/or a functional overload of lymphatics and/or lymph nodes, which leads to the inadequate clearance of lymph. Lymphedema is clinically and histopathologically distinct from common lymphatic malformation and classically involves an entire limb or anatomic region. Lymphedematous tissues slowly enlarge over time because of the accumulation of subcutaneous lymph, which stimulates adipose deposition and fibrosis. Complications of lymphedema include infection, functional disability, chronic cutaneous changes, and psychosocial morbidity [1].

Lymphedema may arise from abnormal development (primary) or injury to lymph nodes or lymphatic vessels (secondary). Secondary lymphedema accounts for more than 90% of patients with the condition worldwide. The most common cause of secondary lymphedema worldwide is parasitic infection. Other causes of secondary lymphedema include treatment for malignancy (lymphadenectomy, radiation, or removal of lymphatics), penetrating trauma, bacterial infection and iatrogenic injury following a surgical procedure to axillary or inguinal lymph nodes [1, 2]. However, secondary lymphedema is rare in the pediatric age group. Approximately 97% of pediatric patients have primary lymphedema [3, 4].

Kaposiform hemangioendothelioma (KHE) is the result of the dysregulation of both angiogenesis and lymphangiogenesis [5, 6]. Lymphedema in patients with KHE is less known and has received sparse attention in the literature, although this complication may be more commonly seen in clinical practice than assumed. Here, we described the clinical features of KHE-associated chronic lymphedema, with the aim of familiarizing specialists with this complication in patients with KHE. In addition, we evaluated the possible association between the use of sirolimus and subsequent lymphedema.

## Methods

### Study patients

Approval was obtained from the Institutional Review Board of West China Hospital of Sichuan University and each participating site. All procedures followed the research protocols approved by West China Hospital of Sichuan University and were conducted according to the Declaration of Helsinki. Written informed consent was provided by the patients or patients’ parents for their clinical records to be used in this study. The KHE patients included in the current study are patients from a cohort consecutively seen in 4 tertiary medical centers between January 2009 and December 2016. The diagnosis of KHE was based on clinical features, magnetic resonance imaging (MRI) and/or histopathological data. In total, 137 patients were diagnosed with KHE during this 8-year interval. These KHE patients had previously been invited to participate in studies of epidemiologic factor, clinical feature, and/or treatment analyses [7–13].

The current study was initiated in 2019 with the aim of obtaining chronic lymphedema information retrospectively. Patients were included in this study if they had a minimum of 3 years of follow-up after the onset of KHE and/or Kasabach–Merritt phenomenon (KMP). In total, 118 patients from the original cohort of 137 were able and willing to participate: 9 patients were unable or unwilling to participate; 6 patients had died, either from KMP or other causes; 4 patients were lost to follow-up.

### Data collection

Interviews conducted by the study investigators assessed patients’ extremity swelling (if any), general physical activity and exercise levels. The diagnosis of chronic lymphedema was based on history, physical examination (e.g., Stemmer’s sign), photographs and lymphoscintigraphic findings, and by consensus of our multidisciplinary consultations dedicated to vascular anomalies. Stemmer’s sign was defined as impossibility to pinch the skin of the dorsal side or the base of the second toe. If extremity swelling occurred during the onset of KHE and/or KMP but did not occur after 1 year, issues relating to lymphedema were not included in this study. Information from medical charts, including demographics, clinical presentations, laboratory results, imaging studies, treatment strategies, and follow-up, were obtained for these 118 patients.

### Definition

The severities of lymphedema were classified as follows: (1) no lymphedema: asymptomatic, no obvious extremity swelling was identified; (2) mild lymphedema: mild symptoms, clinical or diagnostic observations only; (3) moderate lymphedema: moderate symptoms, limiting age-appropriate instrumental activities of daily living (ADL); and 4) severe lymphedema: severe symptoms, limiting self-care ADL. Chronic lymphedema was defined as lymphedema lasting ≥ 6 months after 1 year of the onset of KHE (and/or KMP) [14]. Progression of lymphedema was defined as the enlargement of the affected area or worsening of symptoms and/or complications. According to previous studies, KMP was defined as a platelet count of less than 100 × 10^9^/L, with consumptive coagulopathy and hypofibrinogenemia [9, 15]. Decreased range of motion (ROM) was defined as ≥ 10° of extension loss, flexion < 120°, or both [10].

### Statistical analysis

The data are presented as means with ranges or medians with interquartile ranges (IQRs) for continuous variables and as frequencies with percentages for qualitative variables. The baseline demographics and clinical characteristics were assessed for potential associations with the development of lymphedema. Pearson’s chi-square test and Fisher’s exact test were used to analyze categorical variables. Student’s *t*-test and the Mann–Whitney test were used to analyze continuous variables where appropriate. Statistical analyses were conducted using SPSS 23.0 for Windows (SPSS Inc., Chicago, IL, USA). *P* values less than 0.05 were considered statistically significant.

## Results

### Baseline characteristics

The records of 118 patients with KHE were analyzed. The baseline characteristics of the patients are summarized in Table 1. The mean age at the discovery of the tumor lesion was 19.5 months (range 0.0–576.0 months). The mean age at the diagnosis of KHE was 26.8 months (range 0.0–600.0 months). In total, 74 (62.7%) patients had confirmed KMP. Sixty-seven (56.8%) patients had lesions involving the extremities. Most KHEs were classified as the mixed subtype (72, 61.0%). Thirty-eight (32.2%) patients had lesions ≥ 10.0 cm.

**Table 1:** Demographic and clinical features of KHE patients with or without lymphedema

Variables	Without lymphedema	With lymphedema	Total	*P*^‡^
	*n* = 105	*n* = 13	*n* = 118	
*Patients*				
Sex^†^				0.566^§^
Male	54 (51.4)	8 (61.5)	62 (52.5)	
Female	51 (48.6)	5 (38.4)	56 (47.5)	
Age at discovery of tumor (m)				0.559^#^
Mean (range)	21.3 (0.0–576.0)	5.2 (0.0–12.0)	19.5 (0.0–576.0)	
Median (IQR)	1.0 (0.0–6.8)	3.5 (0.3–7.5)	1.5 (0.0–6.6)	
Age at diagnosis of KHE (m)				0.208^#^
Mean (range)	28.4 (0.0–600.0)	13.5 (0.0–27.0)	26.8 (0.0–600.0)	
Median (IQR)	2.3 (1.0–6.0)	2.1 (1.3–6.1)	2.2 (1.0–6.0)	
KMP^†^				0.366^§^
With KMP	64 (61.0)	10 (76.9)	74 (62.7)	
Without KMP	41 (39.0)	3 (23.1)	44 (37.3)	
*KHE*				
Location^†^				0.006^§^
Extremity involvement	55 (52.4)	13 (100.0)	68 (57.6)	
Non-extremity involvement	50 (47.6)	0 (0)	50 (42.4)	
Morphology^†^				0.009^※^
Superficial	30 (28.6)	0 (0)	30 (25.4)	
Mixed	59 (56.2)	13 (100.0)	72 (61.0)	
Deep	16 (15.2)	0 (0)	16 (13.6)	
Tumor size (cm) ^†^				0.001^§^
< 10 cm	77 (73.3)	3 (23.1)	80 (67.8)	
≥ 10 cm	28 (26.7)	10 (76.9)	38 (32.2)	

*KHE* Kaposiform hemangioendothelioma, *KMP*, Kasabach–Merritt phenomenon, *m* month, *IQR* interquartile range

^†^Values are presented as the number of cases (percentage)

^‡^Patients with lymphedema compared to patients without lymphedema

^§^*P* value was calculated with Fisher’s exact test

^※^*P* value was calculated using the Pearson chi-square test

^#^*P* value was calculated using the Mann–Whitney *U* test

### Lymphedema incidence

Among 118 patients with KHE, none had a family history of primary lymphedema. Chronic lymphedema was confirmed by lymphoscintigraphy in 13 (11.0%) patients after 1 year of the onset of KHE and/or KMP (range 1–5 years). Of these 13 patients, extremity swelling was evident at the presence of KHE (or KMP) in 8 patients (61.5%). In total, 11 (84.6%) patients with lymphedema first experienced extremity swelling within 1 year of the onset of KHE and/or KMP. All patients with lymphedema experienced extremity swelling within 2 years of the onset of KHE and/or KMP.

### Timing of onset of the lymphedema

**Fig. 1:**
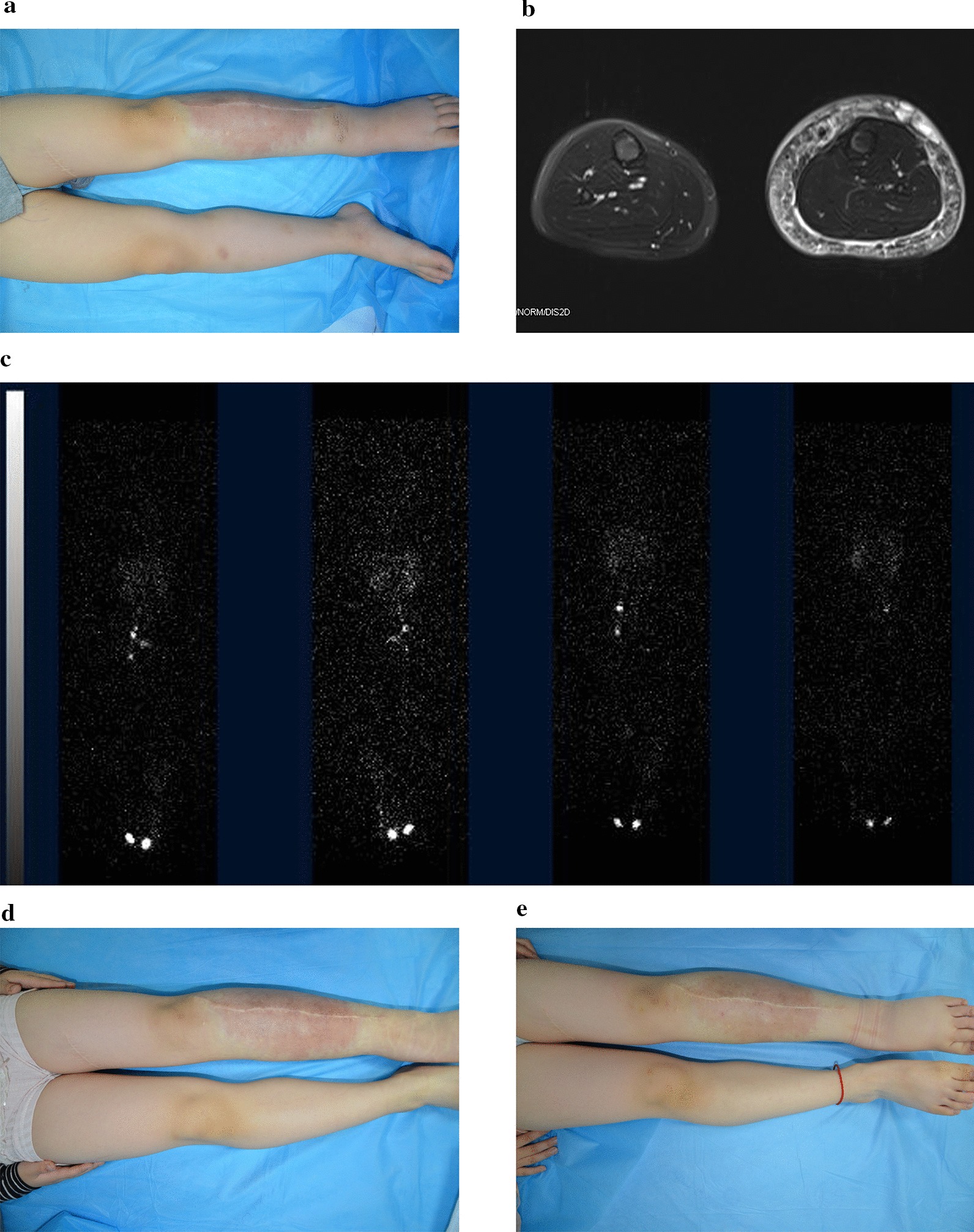
KHEs with chronic lymphedema involving the left lower extremity in a female. This patient’s vascular lesions were noted at 9 months of age. Limb swelling occurred 4 months later. She had partial resection of the thigh and leg KHEs at an outside institution at 2 years of age. **a** The photograph showed an ill-defined hyperpigmented plaque involving the left leg at referral (4 years old). Coronal (**b**) and horizontal (**c**) T2-weighted MRI revealed hyperintense lesions involving the dermis, subcutaneous tissue and deep fascia of the left leg. **d** Nuclear lymphoscintigram obtained 1 and 3 h after pedal injection showed uptake in the right pelvic and inguinal lymph nodes but not on the left side. Note the abnormal accumulation of tracer in the left thigh. **e** The patient received 12 months of sirolimus monotherapy. No alleviation of lymphedema was noted. Then, sirolimus was discontinued, and treatment with compression bandaging was initiated. **g** Twelve months after compression therapy

Two patients were treated before referral (Fig. 1). Of these 2 patients, 1 patient had a congenital lesion with KMP, and had been treated with prednisolone. Although prednisolone improved the associated KMP, the patient suffered from consistent swelling of the involved limb. Another patient was a child who was referred because she presented with progressive swelling of the right leg and foot. She had partial resection of the right thigh and leg KHEs (without KMP) at an outside institution 2 years before referral. In another 7 patients, swelling of the KHE-involved extremity was noted at referral (6 with KMP and 1 without KMP). The remaining 4 patients developed swelling after referral.

**Fig. 2:**
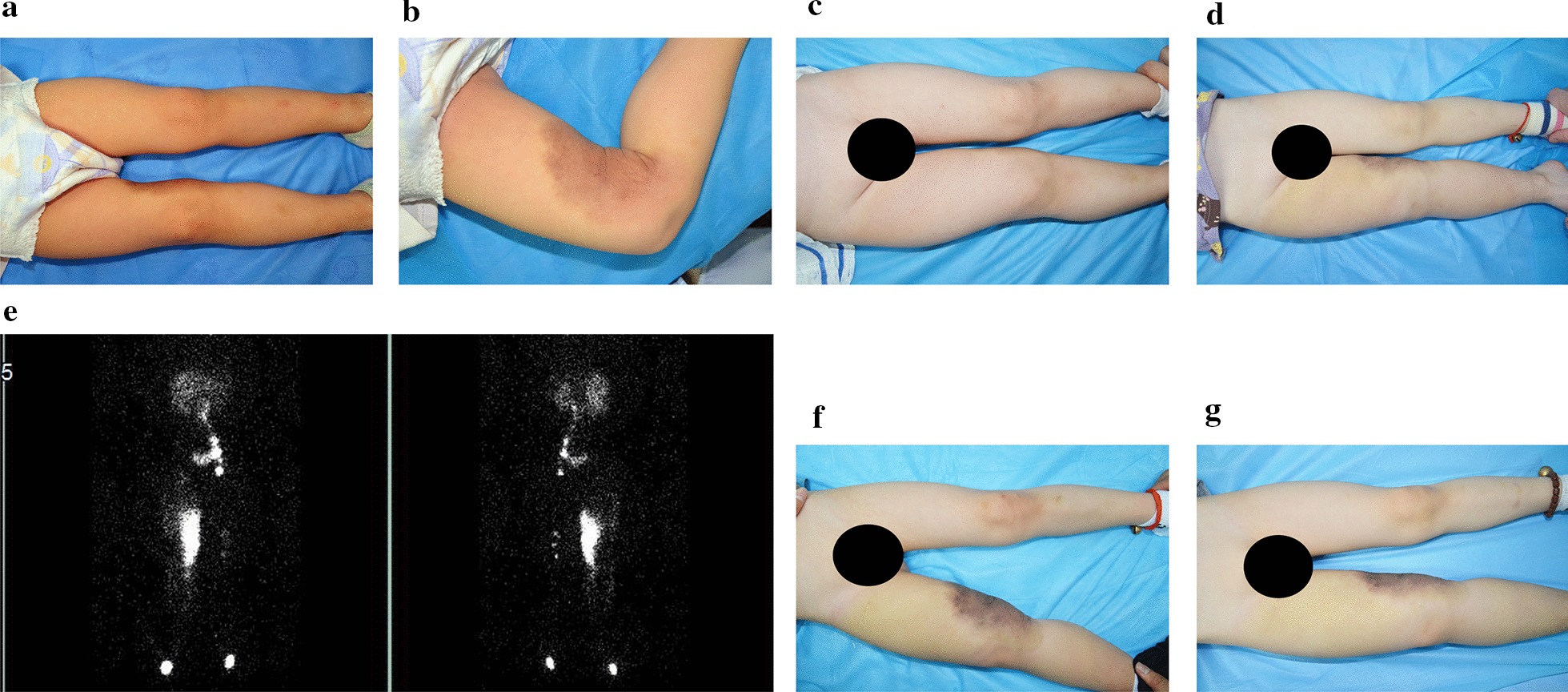
Development of chronic lymphedema in a male with KHE involving the right thigh. **a**, **b** A 2-year-old boy with histopathologically confirmed KHE was treated with sirolimus for 6 months. **c** At 7 months after sirolimus therapy, the photograph showed tight swelling on the right side. **d** At 3 years of age, the photograph showed progressive swelling of the right lower extremity. Sirolimus treatment was then discontinued. **e** Lymphoscintigraphy showed dermal backflow and absent inguinal lymph node drainage 2 h after injection in the right limb. **f** At 3.5-year-old of age, treatment with a compression garment was initiated. **g** Twelve months after compression therapy

Sirolimus was used in 10 (76.9%) patients (versus 67.6% in patients without lymphedema, *P* = 0.369), including 7 with extremity swelling at referral. The remaining 3 patients developed swelling during sirolimus treatment (Fig. 2).

### Characteristics of lymphedema

The mean length of follow-up for patients with lymphedema was 5.1 years (range, 3.2 to 9.0 years). After 1 year of the onset of KHE and/or KMP, 2 (15.4%) patients experienced chronic mild lymphedema only that generally resolved or waxed and waned between none and mild. The remaining 11 (84.6%) patients reported chronic moderate lymphedema. In all patients, lymphedema affected only one extremity, along with ipsilateral KHE. Most (84.6%) lymphedemas involved the lower limb. The involved extremity typically presented nonpitting swelling and was accompanied by noticeable skin changes, mainly thickening and hardening of the skin and subcutaneous tissue. Five (38.5%) patients were considered to have decreased ROM related to lymphedema at the last follow-up. One patient reported repeated cellulitis that was considered related to lymphedema.

### Lymphoscintigraphy

Lymphoscintigraphy demonstrated failure of the superficial collecting lymphatics, resulting in rerouting via small cutaneous collaterals, with a delayed proximal flow of radiolabeled colloid. In 10 patients, no obvious inguinal (8/10) or axillary (2/10) node drainage was observed by lymphoscintigraphy.

### Risk factors for lymphedema

No significant difference was found between patients with lymphedema and patients without lymphedema in terms of sex, age at discovery of the tumor, age at diagnosis of KHE, and presence of KMP. Lymphedema was more likely to be present in patients with mixed lesions. Lesions involving the extremity were more likely to have lymphedema than the non-extremity lesions. Additionally, compared with patients whose lesions were < 10 cm, those whose lesions were ≥ 10 cm were more likely to experience lymphedema.

### Treatments

The management and treatment protocols for KHE were variable in the entire group of 118 study subjects. In all, 66 (55.9%) patients received corticosteroids (orally, intravenously, or intramuscularly administered). Conventional drugs, including propranolol and vincristine, and surgical excision were also used. Since 2011, sirolimus plus corticosteroid and sirolimus monotherapy have been the most common treatment regimens for KHE with KMP and KHE without KMP, respectively.

**Fig. 3:**
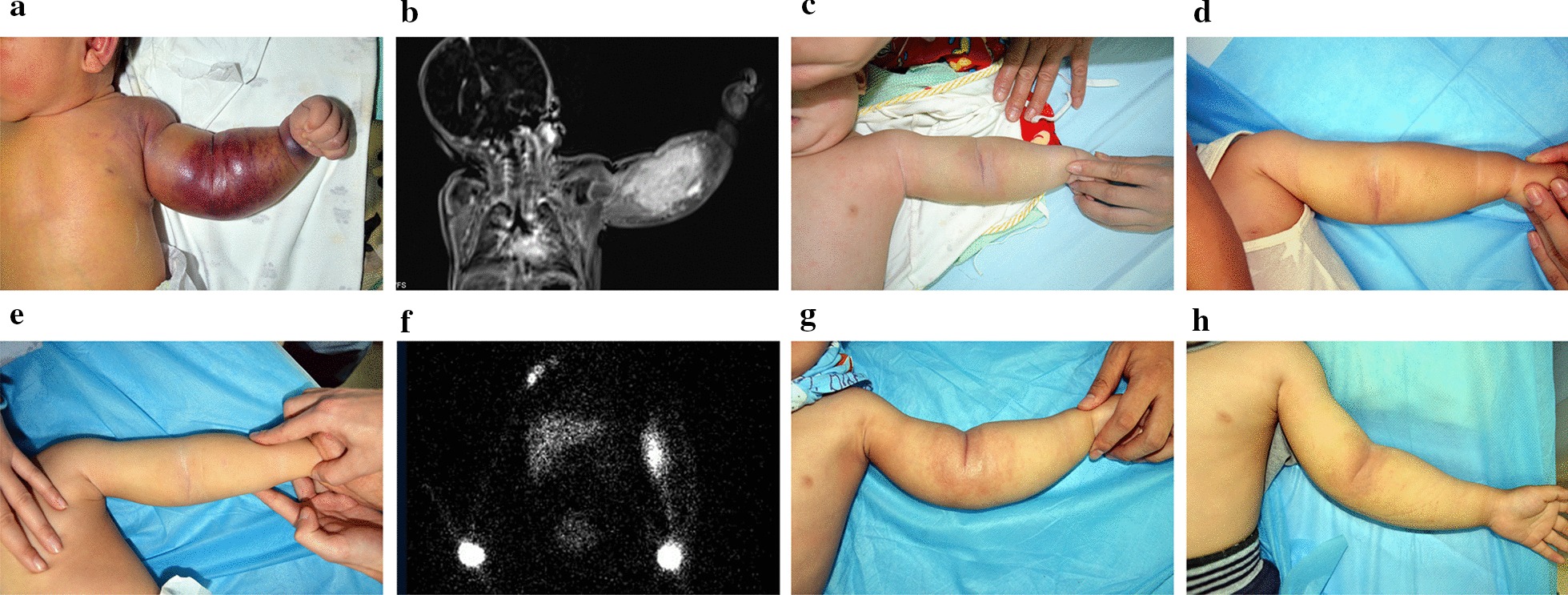
Development of chronic lymphedema in a male with congenital KHE with KMP. **a** A 2-day-old male infant was referred due to a congenital vascular mass on his left upper extremity. The mass was indurate and purpuric. His platelet count was 9 × 10 [9]/L. **b** Coronal T2-weighted MRI revealed a hyperintense left upper extremity mass with ill-defined margins and reticular stranding in multiple tissue planes. Photographs at 6 (**c**), 12 (**d**) and 18 (**e**) months after sirolimus treatment showed a decrease in extremity swelling. **f** At 2 years of age, lymphoscintigraphy demonstrated the absence of draining tracts and axillary node drainage 2 h after injection, with backflow in cutaneous collateral lymphatics. A diagnosis of lymphedema was made. The sirolimus therapy was tapered and discontinued. **g** Three months after the cessation of sirolimus; the photograph showed a rebound of the extremity swelling, requiring sirolimus reintroduction. **h** At 3.5 years of age, the patient still received a low dose of sirolimus (trough level, 3–5 ng/ml)

All patients with lymphedema received at least one type of treatment. In 7 patients with extremity swelling at referral, although they showed significant improvement of the extremity swelling after sirolimus treatment, lymphedema persisted during the follow-up periods (Fig. 3). In 3 patients, lymphedema showed progression despite prolonged sirolimus treatment, and no improvement in lymphedema was identified after the discontinuation of sirolimus. Compression therapy using custom-fitted garments or bandaging was prescribed in 7 patients. None of the patients had surgical management for lymphedema during the follow-up period. None of the patients showed complete lymphedema eradication at their last follow-up.

## Discussion

Swelling of the KHE-affected area was common after the onset of KHE, especially during the presence of KMP [9, 15]. Because of the infrequent use of lymphoscintigraphy for confirmatory imaging, the actual prevalence and incidence of lymphedema in patients with KHE are most likely higher than those indicated in the limited published reports [16, 17]. The current study aimed to evaluate practical questions about lymphedema in long-term follow-up subsequent to the onset of KHE and/or KMP. We found that the incidence of chronic lymphedema in patients with KHE was high: 11.0% had chronic lymphedema after 1 year of the onset of KHE and/or KMP.

Remarkably, lymphedema can be an unusual complication of sirolimus therapy. The development of lymphedema in KHE patients after sirolimus treatment has been reported previously [17, 18]. Some investigators speculated that lymphedema was the result of sirolimus therapy itself [17]. There are studies of sirolimus-associated lymphedema of the upper and lower limbs in the transplantation literature [19–21]. However, in contrast to our observation in KHE, some patients with sirolimus-associated lymphedema usually showed improvement or resolution of lymphedema after discontinuing sirolimus. More than half of our patients had visible extremity swelling before sirolimus treatment. Some of our patients with lymphedema had never been treated with sirolimus. Our data did not support the concept of a relationship between sirolimus and lymphedema onset in patients with KHE. In addition, variable responses to sirolimus treatment of lymphedema have been noted in our patients. Most importantly, the affected lymphedema site was anatomically related to the site of KHE. There was a clear spatiotemporal relationship between KHE (or KMP) and lymphedema onset. No bilateral lymphedema was observed.

There is no generally accepted explanation of the pathophysiology of lymphedema in patients with KHE. The observation of lymphedema formation with KHE indicates the interference of this disease with lymph drainage. Lymphangiogenesis is the main pathological feature of both KHE and lymphedema [5, 6, 22]. In addition to expressing vascular endothelial growth factor receptor-3 (VEGFR-3), KHE-derived mesenchymal stem cells also show higher levels of vascular endothelial growth factor-C (VEGF-C) than normal lymphatic endothelial cells [23]. The VEGF-C/VEGFR-3 signaling pathway is important in modulating lymphangiogenesis and the immune response. Constant stimulation of VEGF-C/VEGFR-3 signaling is required for maintaining lymphatic vessel plasticity and stability. The disruption of VEGF-C/VEGFR-3 signaling can completely destroy the lymphatic network and lead to a lymphedema-like phenotype [22]. Therefore, the involution of KHE, together with the decreased activity of VEGF-C/VEGFR-3 signaling, may remodel lymphangiogenesis. This may be a potential explanation for the development of lymphedema in patients with KHE.

There may also be alternative mechanisms by which KHE induces lymphedema. The infiltration of the KHE to local lymphatics and subsequent fibrosis may disrupt lymphatic flow of the affected limbs. In most patients with KHE, the tumor was still present even after long-term treatment, and the lesion was characterized by progressive fibrosis [24]. This may explain the phenomenon that lymphedema can present months or years after the onset of KHE and/or KMP. In addition, inflammation with KMP may cause the blockage and reflux of a multitude of lymphatics [6]. These factors could explain lymphedema within the same lymph drainage territory as KHE. It is reasonable that more extensive KHE (large and mixed type) results in more extensive disruption of lymphatic vessels and, consequently, is associated with an increased risk of lymphedema formation. Once lymphedema is established, the subsequent pathophysiology may be independent of its cause (e.g., KHE).

Clinically, lymphoscintigraphy is the gold standard imaging study for the diagnosis of lymphedema and quantitative assessment of lymphatic function. Although limited by poor anatomic definition, lymphoscintigraphy is possible by utilizing a multitude of isotopic agents and is not associated with morbidity [1]. Reliable functional information on the lymphatic systems can be obtained using this simple and safe method. However, although MRI is less specific than lymphoscintigraphy in distinguishing lymphedema from other vascular anomalies, MRI has value in the diagnosis of KHE [3, 25]. In addition, MRI can clearly determine the extent of tumor involvement and response to treatment [26].

Although rare, infections (e.g., cellulitis) can occur in KHE patients with lymphedema. We also found that nearly half of our lymphedema patients had fluctuating or increased swelling or symptoms (e.g., functional limitations) despite treatment with sirolimus. Given the rarity and high morbidity of this condition, KHE in combination with lymphedema is best managed by an interdisciplinary team focused on vascular anomalies. In our cohort, compression was the mainstay of management of lymphedema other than oral sirolimus. Theoretically, the compression of lymphedematous tissue decreases the size of the area by reducing the amount of edema and slows the progression of the disease by reducing the adipose deposition that results from high-protein fluid in the interstitium.

It is important to consider the limitations as well as the strengths of the study. First, the medical information may have been affected by the retrospective nature of our data collection. A selection bias existed because patients and parents concerned about health status were more likely to participate in the study. Therefore, the medical information may suffer from possible recall bias and underestimation of the incidence of lymphedema. Second, the risk factor analyses did not take into account multiple comparisons. Therefore, some significant relationships may be spurious. Our findings need to be confirmed in future investigations. Third, the lack of a clear standard definition of chronic lymphedema increases the difficulty of investigating the clinical features and determining the accurate prevalence of this rare disorder. Finally, our patients received various types of treatment regimens. We cannot exclude the possibility that treatment regimens have an effect on the development of lymphedema. Whether oral sirolimus can facilitate lymphedema in patients with KHE requires further confirmation. However, the strengths of the study include its large sample size and long-term follow-up. In addition, the study provides the most extensive and detailed information on lymphedema associated with KHE. A similar prospective study is presently not possible because of the relative rarity of this condition.

## Conclusions

Chronic lymphedema is a long-term sequela of KHE, and typically occurs along with KHE in the ipsilateral extremity. Patients with large and mixed KHE involving extremities should be closely monitored for this disabling complication. Chronic lymphedema in patients with KHE seems to be irreversible and resistant to sirolimus treatment. Better treatments are needed to improve the life expectancy of patients with KHE and associated chronic lymphedema.

## Data Availability

The datasets used and/or analyzed during the current study available from the corresponding author on reasonable request.

## Abbreviations

KHE: Kaposiform hemangioendothelioma
KMP: Kasabach–Merritt phenomenon
CT: Computed tomography
MRI: Magnetic resonance imaging
ADL: Activities of daily living
ROM: Decreased range of motion
VEGFR-3: Vascular endothelial growth factor receptor-3
VEGF-C: Vascular endothelial growth factor-C
